# Tailored Informational Interventions for Reducing Surplus and Waste of Fruits and Vegetables in a Food Market: A Pilot Study

**DOI:** 10.3390/foods12122313

**Published:** 2023-06-08

**Authors:** Carolina Fredes, María Ignacia Pérez, Macarena Jimenez, Beatriz Reutter, Rodrigo Fernández-Verdejo

**Affiliations:** 1 Departamento Ciencias de la Salud, Carrera de Nutrición y Dietética, Facultad de Medicina, Pontificia Universidad Católica de Chile, Santiago 7820436, Chile; mcperez7@uc.cl; 2 Instituto para el Desarrollo Sustentable, Pontificia Universidad Católica de Chile, Santiago 7820436, Chile; majimenez@uc.cl; 3 Dirección Regional de la FAO para América Latina y el Caribe (RLC), Santiago 7630412, Chile; beatriz.reuttersusaeta@fao.org; 4 Laboratorio de Fisiología del Ejercicio y Metabolismo (LABFEM), Escuela de Kinesiología, Facultad de Medicina, Universidad Finis Terrae, Santiago 7501015, Chile

**Keywords:** food waste, food surplus, behavioral intervention, food distribution

## Abstract

This pilot study explored the effectiveness of tailored informational interventions to reduce the surplus and waste of fruits and vegetables at the distribution level in Chile. Stalls from a fresh food market were randomized to intervention (*n* = 5 selling fruits, *n* = 5 selling vegetables) or control (*n* = 4 selling fruits, *n* = 4 selling vegetables) groups. The causes of surplus and waste were estimated by questionnaires. Surplus, avoidable waste, and unavoidable waste were measured using direct quantification before and after the intervention, and were expressed relative to the initial stock. Before the intervention, the surplus was (median [25th–75th percentile]) 46.2% [33.3–51.2] for fruits and 51.5% [41.3–55.0] for vegetables; avoidable waste was 0.1% [0.0–0.8] for fruits and 1.8% [0.7–5.3] for vegetables; and unavoidable waste was 0.0% [0.0–1.0] for fruits and 0.0% [0.0–1.3] for vegetables. Planning and storage represented the main causes explaining surplus and waste. After the intervention, the intervention group decreased the surplus of fruits compared to the control group (−17.8% [−29.0–−11.0] vs. 5.8% [−0.6–7.8], respectively; *p* = 0.016), without other differences. In conclusion, tailored informational interventions based on the causes of surplus and waste may reduce the surplus of fruits in a fresh food market. Interventions might also include management strategies for the surplus to improve grocers’ business operations.

## 1. Introduction

Food waste represents food that is fit for human consumption but has been removed from the food supply chain at the distribution or consumption stages [1]. Previous data showed that 35–55% of fruits and vegetables are lost or wasted globally [2]. In addition to food waste, food surplus often occurs along the food supply chain [3,4]. The surplus of fruits and vegetables is usually discarded due to oversupply or aesthetic criteria [5,6]. However, notably, this surplus maintains its nutritional and organoleptic properties, and could thus be redistributed to the food industry (e.g., to produce fruit juice) or people in need [7]. Redistribution and donation to food banks, soup kitchens, and shelters may help increase the access of vulnerable people to fruits and vegetables, and reduce food waste [8,9,10,11,12]. Therefore, planning actions aimed at preventing or reducing food waste should tackle food waste itself and also food surplus [3,13].

Globally, the fruit and vegetable sector provides fresh foods with great nutritional value to consumers [14]. The international trade of fresh fruits and vegetables represents only ~7–8% of the total global production. Nevertheless, such a trade exchanges 758 million tons of fruits and 1256 million tons of vegetables along the food supply chain (calculated from [15]). Fresh food markets thus handle large quantities of fruits and vegetables [5]. Because fruits and vegetables are perishable, they are at risk of ending up uneaten [1]. This perishability may partly explain why ~10% of the initial production of fruit and vegetable at the distribution level (e.g., wholesale markets and retail) ends up as waste [2].

The production of fruits and vegetables in Chile is concentrated in the central regions (e.g., Metropolitan Region), including 2.5 million tons of fruits and 6.7 million tons of vegetables (calculated from [15]). According to the National Registry of fresh food markets, there are 1114 markets in Chile, gathering 113,112 stalls and 340,000 grocers [16]. Notably, 81% of those stalls sell fruits, vegetables, or both [16]. In Chile, the high coverage of fresh food markets favors the physical and economic access to fruits and vegetables of the low-income population [17]. At the same time, fresh food markets face the challenge of managing the waste of foods with great nutritional value. Suitable management at the stalls would allow grocers to improve their commercial operation and reduce food surplus and avoidable food waste. For example, management could focus on avoiding overstock that usually becomes avoidable waste, and this would also decrease purchase and handling costs. Suitable management could also benefit consumers through the redistribution of food surplus, thus helping them face food insecurity [18]. The Food-Based Dietary Guidelines recommend consuming at least five servings of fruits and vegetables daily [19]. Yet, only 40% of people with a low income meet this recommendation in Chile [20]. Thus, the design of effective interventions to reduce fruit and vegetable waste in fresh food markets has environmental, social, and nutritional implications.

Food loss and waste have several causes along the food supply chain [2,21], with some of them being pertinent to fresh food markets. In developing countries, food loss and waste occur mostly at production and postharvest handling stages, whereas in developed countries, they occur mostly at the consumption level [2]. The highest food loss in developing countries can result from a lack of technology and infrastructure along with poor knowledge of stakeholders [1,2,7,22]. In low- and middle-income countries, the poor functioning of the supply chain mostly explains the food loss by smallholders [7]. In developed countries, farmers report cosmetic specifications by retailers and a lack of processing facilities as causes of food waste [23]. Note, however, that farmers consider food loss to be a non-priority problem and perceive food loss as an intrinsic part of the business operation at the production level [23]. Another cause of food loss is poor bulk packaging during transport [7]. In developing countries, a lack of refrigeration infrastructure also explains the food waste at the distribution stage [2,22]. Yeasts and molds thrive on unrefrigerated fruits and vegetables, promoting them to spoil quickly in hot, humid climates, thus limiting their postharvest. In fresh food markets, physical conditions (e.g., temperature and humidity) may also affect postharvest life and product appearance [21]. At the distribution and consumer levels, the usual causes of waste include inappropriate (or non-existent) storage conditions, excessive handling of fruit and vegetables, and behavioral issues of grocers and consumers (e.g., discarding fruits and vegetables because of aesthetic reasons) [7,21,24,25]. Note that some fruits and vegetables have an inedible fraction or a fraction perceived to be inedible based on the cultural preferences of consumers [3,26,27]. Due to the various determinants of food surplus and waste in the fruit and vegetable sector, identifying the main causes of food waste is essential to design tailored interventions [5,28].

From the perspective of food waste, interventions represent particular actions applied on food systems aimed at reducing food waste [29]. Informational interventions are the most common to prevent food waste, and may represent a first approach to creating awareness and promoting behavioral changes associated with food waste [28,30,31,32,33]. Informational interventions involve strategies through different communication channels to increase the knowledge and skills of stakeholders along the food supply chain [30,31,32]. Nevertheless, there is scarce evidence on the effectiveness of informational interventions [30].

To gain insight into this topic, we delivered informational interventions to reduce the surplus and waste of fruits and vegetables in the stalls of a fresh food market. The interventions were tailored based on the causes of surplus and waste reported by the grocers. The aim of this pilot study was to explore the effectiveness of tailored informational interventions to reduce the surplus and waste of fruits and vegetables at the stalls.

## 2. Materials and Methods

### 2.1. Fresh Food Market and Stalls

The research was conducted at the fresh food market “El Feriante” in the Estación Central district, Santiago (Metropolitan Region, Chile). The market has 73 stalls that operate on Tuesdays and Fridays from 07:00 to 15:00. Fifty-six stalls sell fruits, vegetables, or both. Due to the COVID-19 pandemic, the stalls were partially open on Tuesdays. Therefore, we conducted all the measurements on Fridays during July and August 2021; mean air temperature ranged between 0 and 15 °C. The grocers buy fruits and vegetables from two wholesale markets located in Santiago. Neither the wholesale markets nor the fresh food market had refrigeration infrastructure. Prior to the measurement period, the procedures were explained to interested grocers. Each grocer who agreed to participate in the research signed an informed consent form. This research was approved by the Ethics Committee of the Pontificia Universidad Católica de Chile (ID 210311009). Grocers were not part of other interventions or campaigns to prevent food waste for the duration of the study.

Note that the usual waste management at the fresh food market requires grocers to deposit the waste into bags (without any separation), containers, or adjacent land. The waste is then collected by a municipal truck managed by the environment department of the Estación Central district. Finally, the waste is disposed of in landfill (0% CH_4_ capture) without any treatment. 

### 2.2. Design

**Figure 1:**
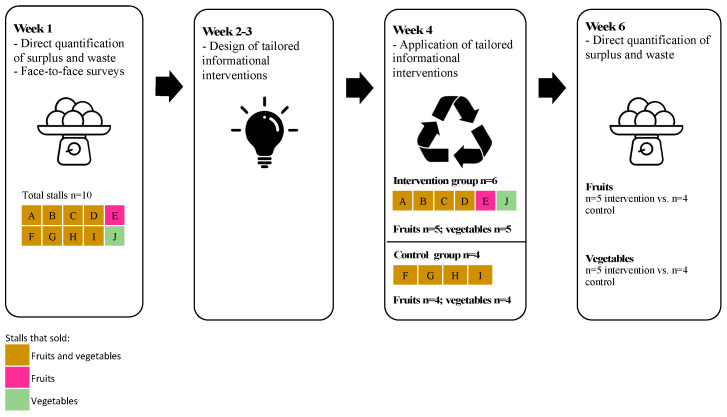
Study design.

This was a pilot study with an experimental, randomized, controlled, and repeated measures design [34]. Figure 1 summarizes the design. Baseline measurements (“before” time point) were conducted by teams comprised of one Researcher (either C.F., M.I.P., or M.J.) and 4–5 undergraduate students from the course “Food Loss and Waste” using Service-Learning projects at Pontificia Universidad Católica de Chile. Each team quantified the surplus and waste of fruits and vegetables of two stalls in one day. Ten stalls were measured in total; eight sold both fruits and vegetables, one only fruits, and one only vegetables. Therefore, nine stalls sold fruits and nine sold vegetables. A structured face-to-face survey was also applied to grocers to identify the reasons to explain the waste of that day. Table 1 summarizes the sociodemographic characteristics of the grocers. Note that all grocers had, at least, a basic level for using technological communications tools such as smartphones and web conferencing tools.

Stalls were randomly assigned to an intervention or control group, verifying that the stalls of the control group were not physically adjacent to the stalls of the intervention group. Based on the results of the survey, we designed informational interventions to reduce surplus and waste tailored to the stalls of the intervention group. The informational interventions were delivered to the intervention group, whereas the control group had no intervention. Two weeks later, the quantification of surplus and waste was repeated in all stalls (“after” time point).

### 2.3. Direct Quantification of Fruit and Vegetable Surplus and Waste

Unsold fruits and vegetables during the measurement day were considered as surplus. We consider the FUSIONS definition of food waste [35]. Therefore, we quantified fruits and vegetables, and their inedible parts, that were disposed of after cleaning, handling, and selling at stalls. Direct weighing was used for quantification. Briefly, at the beginning of the measurement day, all fruits and vegetables available for commercialization were counted and/or weighed on an electronic scale (Seca 813, SECA, Hamburg, Germany; capacity 200 kg, graduation 100 g). This was considered the initial stock. We provided four plastic containers per stall to dispose of: (a) avoidable fruit waste, (b) avoidable vegetable waste, (c) unavoidable fruit waste, and (d) unavoidable vegetable waste. Therefore, the initial stock is represented by:

Initial stock=sold products+surplus+avoidable waste+unavoidable waste

**Figure 2:**
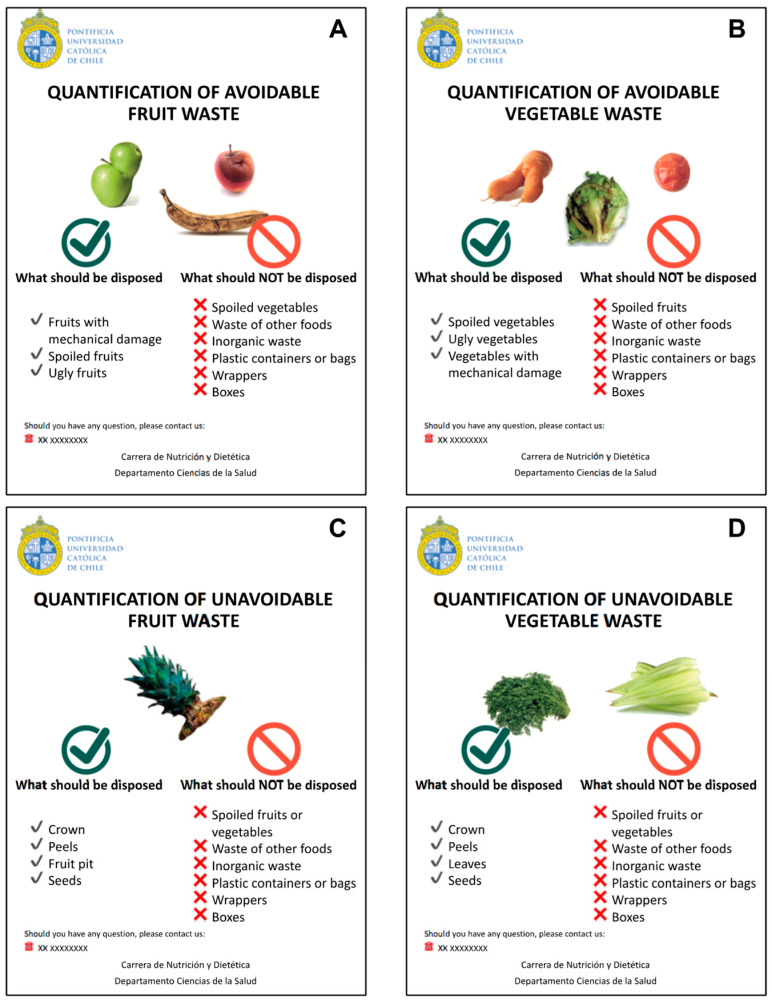
Infographics displayed in stalls to guide grocers on the disposal of (**A**) avoidable fruit waste, (**B**) avoidable vegetable waste, (**C**) unavoidable fruit waste, and (**D**) unavoidable vegetable waste.

Each container had an infographic that detailed what should and should not be disposed of (Figure 2). Specifically, avoidable waste included deteriorated (e.g., mechanically damaged or spoiled) and “ugly” fruits and vegetables that were discarded; unavoidable waste included inedible parts of fruits and vegetables that were discarded (e.g., crown, fruit pit, leaves, and stems). This procedure was performed at the “before” and “after” time points. To minimize desirability bias, all research procedures were explained to the grocers emphasizing that fruit and vegetable wasting could be an inherent part of the process of selling the products. 

At the end of the measurement day, the waste within each container was revised and weighed on the electronic scale. Since we did not separate the waste within each container, the categories of fruit (e.g., citrus and pomaces) and vegetable (e.g., leafy vegetables and bulbs) could not be distinguished. The surplus was also counted and/or weighed on an electronic scale. The percentages of surplus, avoidable waste, and unavoidable waste of each stall were calculated for fruits and vegetables separately as:

$$\mathrm{Surplus}\;(\%)=\frac{\mathrm{Surplus}\;(\mathrm{kg})}{\mathrm{Initial}\;\mathrm{stock}\;(\mathrm{kg})}\;\times \;100$$

$$\mathrm{Avoidable}\;\mathrm{waste}\;(\%)=\frac{\mathrm{Avoidable}\;\mathrm{waste}\;(\mathrm{kg})}{\mathrm{Initial}\;\mathrm{stock}\;(\mathrm{kg})}\;\times \;100$$

$$\mathrm{Unavoidable}\;\mathrm{waste}\;(\%)=\frac{\mathrm{Unavoidable}\;\mathrm{waste}\;(\mathrm{kg})}{\mathrm{Initial}\;\mathrm{stock}\;(\mathrm{kg})}\;\times \;100$$

### 2.4. Financial Value and Nutritional Content of Avoidable Waste 

Each fruit and vegetable in the initial stock was identified, and its price was retrieved from a database of wholesale markets products [36]. We averaged the price of all the fruits, thus obtaining (mean [minimum–maximum]) 1.94 [1.48–2.05] USD/kg of fruit. For vegetables sold as USD/unit, we converted the price to USD/g. Thus, for vegetables, the average price was 1.73 [1.65–1.98] USD/kg. These average prices were used to compute the financial value of the avoidable waste of fruits and vegetables within each stall.

Similarly, the nutritional content (i.e., energy, carbohydrate, dietary fiber, vitamin C, and potassium) of fruits and vegetables identified in the initial stock was retrieved form FoodData Central database [37]. We then averaged the content of energy and nutrients per gram of fruits and vegetables. These average values were used to compute the nutritional content of the avoidable waste of fruits and vegetables within each stall. We did not consider protein (nitrogen) content because fresh fruits and vegetables have rather low protein contents (0 to 2 g/portion) [37].

### 2.5. Survey to Explore Reasons that Explain Surplus and Waste of Fruits and Vegetables

The survey was adapted from published surveys [12,38,39,40] considering reasons that apply to perishable products such as fruits and vegetables. The survey included four types of reasons within each category of fruits and vegetables associated with the determinants of surplus and waste. The reasons were thus associated with: (a) grocer planning, including overbuying, purchasing without a sales plan, lack of staff to serve customers, overstock, and few customers during the day; (b) product storage, including high perishability of products, product does not come refrigerated, lack of refrigeration capacity at the fresh food market, and inadequate cooling at the stall; (c) stall conditions, including direct exposure to sunlight of the products, excess humidity, poor ventilation, and lack of space for products; and (d) product handling, including products looking dehydrated and mechanical damage of products. These four categories of reasons (i.e., planning, storage, stalls, and handling) then constituted the pillars of the tailored informational interventions.

Nine categories of products were considered (Table 2), according to their availability in the Metropolitan Region [36]. For each category, grocers could reply whether or not they recognized each of the possible reasons for surplus and/or waste at their stalls (i.e., single choice yes/no for each reason category). Grocers could abstain if they were unsure and could add comments if necessary. The results of the survey were expressed as the percentage of grocers that recognized (i.e., responded “yes”) each reason for each fruit and vegetable category.

Note that the measurements were conducted in Santiago during winter. However, the reasons that explain the surplus and waste of fruits and vegetables may be different in other cities and seasons. We took into consideration this issue in the tailored informational intervention by including recommendations for other seasons.

### 2.6. Tailored Informational Intervention

The informational interventions were aimed at improving the knowledge of grocers about planning, storage, stall conditions, and product handling to create awareness and promote behavioral changes associated with food waste. Recommendations for each pillar of intervention (i.e., planning, storage, stalls, and handling) were constructed based on the previous literature on interventions to prevent food waste [28,33,41,42,43].

Tailored informational interventions were designed based on the results of the survey. The informational intervention thus entailed recommendations to improve one or another pillar of intervention according to the main reasons that explained the surplus and waste reported by each grocer. For example, if a grocer reported overstock and few customers during the day as reasons (i.e., planning issues), the tailored informational intervention was focused on recommendations to improve planning (e.g., to reduce initial stock). If a grocer reported positive actions that prevent surplus or waste, we reinforced those actions as good practices. Since the causes of surplus and waste may vary between seasons (e.g., direct exposure to sunlight and excess humidity), we also included recommendations for the spring and summer months. 

Tailored informational interventions were delivered individually to each grocer in the intervention group via a videoconference three weeks after the baseline measurements. During the videoconference, each grocer could ask any questions. After the videoconference, a summary file with the recommendations for each pillar was delivered to each grocer via smartphone messaging to reinforce the recommendations. One week later, each grocer was contacted to arrange a second visit to the stall (“after” time point).

### 2.7. Sample Size

As a pilot study, we had no preliminary data available. Sample size calculation was thus not possible [44,45]. However, the data obtained from this study will allow sample size calculations in future studies. We included five stalls per group based on feasibility. We then calculated the sensitivity to detect differences between the groups using the software G*Power version 3. With five stalls per group, using the Mann–Whitney U test, an α of 5%, and a β of 20%, we were able to detect an effect size (difference/standard deviation) of 2.27. This represents a difference between stalls equivalent to 2.27 times the variability (standard deviation) of the measurement. Consequently, the study was only powered to detect rather large differences between groups. If there were actual differences but of a smaller magnitude between groups (i.e., effect size lower than 2.27), we were unpowered to detect them.

### 2.8. Statistics

Considering the small sample size, non-parametric analyses were used throughout. Data for categorical variables were presented as frequency (percentage). Fischer’s exact test (two-sided) was used to determine the association between each reason to explain waste/surplus (yes or no, for each reason category) and the group (control, intervention). Data for continuous variables were presented as median [25th percentile–75th percentile]. The change (Δ) in surplus, avoidable waste, and unavoidable waste in response to the intervention was computed as: After (%)–Before (%). Mann–Whitney U test was used to compare distributions of ΔSurplus, ΔAvoidable waste, and ΔUnavoidable waste between groups (control, intervention). IBM^®^ SPSS Statistics version 27 was used, considering a *p*-value < 0.05 as statistically significant.

## 3. Results

### 3.1. Reasons to Explain Surplus and Waste at Stalls

Table 3 presents the reasons reported to explain waste before the intervention. There were no differences between the groups. Notably, among the fruit categories, all stalls reported planning and storage as reasons to explain waste. Among the vegetable categories, all stalls reported storage capacity as a reason, and ≥80% of stalls reported planning. Conditions within the stalls and handling were, in general, less reported for most fruit and vegetable categories. Therefore, the design of tailored informational interventions was mostly focused on improving planning and storage. Table 4 summarizes recommendations for each pillar used in tailored informational interventions.

### 3.2. Changes in Response to Interventions

**Figure 3:**
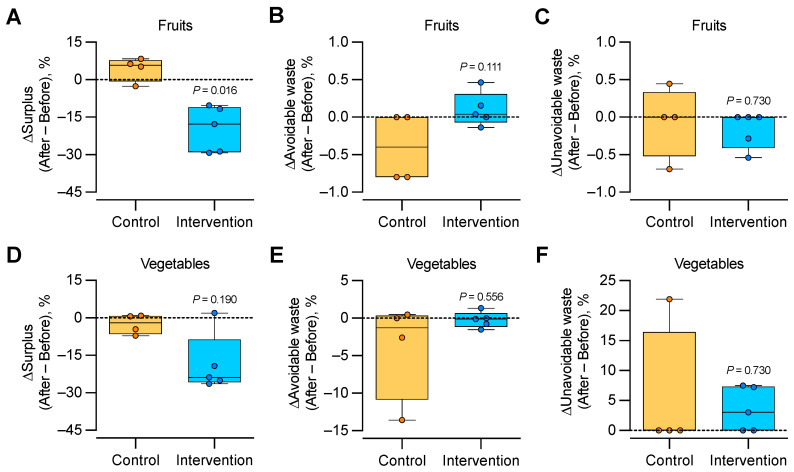
ΔSurplus, ΔAvoidable waste, and ΔUnavoidable waste of fruits (**A**–**C**) and vegetables (**D**–**F**) at stalls. Boxes represent the 25th and 75th percentiles, with a horizontal line denoting the median; whiskers denote the minimum and maximum values. Circles represent raw data points. Mann–Whitney U Test was used for comparison between groups.

Table 5 shows the descriptive data for fruit and vegetable stock, surplus and waste, and the financial value and nutritional content of avoidable waste before and after the intervention. The groups had no difference in any variable before the intervention (*p* ≥ 0.190 in all cases, Mann–Whitney U test). Following the intervention, all stalls in the intervention group reduced their fruit surplus, and therefore, ΔSurplus was lower in the intervention than in the control group (Figure 3A). In contrast, the groups had no difference in the ΔAvoidable waste or ΔUnavoidable waste of fruits (Figure 3B,C), nor did they have a difference in the ΔSurplus, ΔAvoidable waste, or ΔUnavoidable waste of vegetables (Figure 3D–F).

## 4. Discussion

Prevention has been highlighted as the best strategy to reduce food waste from an environmental standpoint [47,48]. Herein, we designed a randomized controlled pilot study to gain insight into the tailored informational interventions and to explore their possible effectiveness to reduce surplus and waste of fruits and vegetables. Our results suggested that tailored informational interventions—focused on planning and storage—reduce fruit surplus at stalls in a fresh food market. This is especially relevant at the distribution stage, as unsold fruits and vegetables most probably become avoidable waste [49], carrying economic and nutritional losses. In this section, we first discuss the causes that explained surplus and waste at the stalls before the intervention, and how those causes influenced the design of the tailored informational interventions. We then discuss the effectiveness of tailored informational interventions in reducing surplus and waste. Finally, we comment on the economic cost and nutritional content of the avoidable waste.

### 4.1. Reasons That Explained Surplus and Waste, and Their Influence on Tailored Informational Interventions

The reasons that explained the waste of fruits and vegetables were mainly associated with planning and storage. Grocers reported overstock and having few customers during the day as the main reasons. These reasons directly affect the grocer’s planning. Due to the COVID-19 pandemic, business activities were either locked or partially operational, and people (the customers) had movement restrictions [50]. Grocers could thus struggle to manage the amount of stock (e.g., one vs. two days of selling per week) and/or have uncertainty about the number of customers (e.g., customers that preferred not to visit the market). Consequently, recommendations provided to grocers as part of the informational interventions were focused on reducing the excessive stock [42] and selling the surplus at a reduced price along the day [41]. Note that grocers (*n* = 4) denoted selling the surplus at a reduced price at the end of the operation day. Nevertheless, they did not express concerns regarding the financial implications of this practice. In contrast, some grocers (*n* = 6) preferred not to sell the surplus and stored it with the uncertainty of whether they would be able to sell it on the next operation day. Future studies should address the financial implications of either selling the surplus at a reduced price or storing the surplus for the next operation day. Reuse and recycling are less preferred strategies in waste or food recovery hierarchies compared to reducing the source of surplus [13,22,48,51]. Nevertheless, we also provided recommendations to grocers about reducing and recycling, for example adhering to redistribution programs [46]. Note that redistribution of the surplus to soup kitchens and shelters may not be an attractive strategy for the grocers to implement. This is because the grocers are not directly benefited. Grocers commented that they understood the social and environmental implications of redistribution programs, but they did not see economic benefits in their business operations. Therefore, future interventions associated with the redistribution of surpluses should evaluate possible economic implications for grocers. Redistributing waste for animal feeding [42] was also included as a recommendation. This recommendation would be more feasible since it does not imply an economic loss for the grocers. Some grocers commented that they informally redistribute the waste of fruits and vegetables to help feed animals. Future interventions at this level could include a waste collection center at the fresh food market to facilitate the logistics of recovery for redistribution to animal feed.

Reasons associated with product storage reported by grocers result from the lack of refrigeration infrastructure at the fresh market. For example, products that do not come refrigerated and lack refrigeration capacity at the fresh food market were frequently reported. The lack of infrastructure has been extensively reported as a determinant of food waste, especially for fresh fruits and vegetables [2,7,21]. The use of adequate technology and infrastructure is essential to reduce food loss and waste by increasing efficiency and productivity in food systems associated with fruits and vegetables [1]. Note that fresh fruits and vegetables are highly perishable products with a limited shelf-life [37,52]. Grocers then have to manage complex logistics to ensure the food quality and food safety. An adequate chilling and cold storage of fruits and vegetables enhances postharvest life, thus increasing shelf-lives during commercialization [53]. Without refrigeration infrastructure at the fresh food market, the marketing period for grocers is shorter because of a shorter postharvest of fruits and vegetables. Yet, the improvement in the refrigeration infrastructure involves other types of interventions. Therefore, we delivered more feasible storage recommendations, such as maintaining the cold chain before and after the day of operation [42], and separating storage for climacteric and non-climacteric products [41]. 

Note that our study was conducted in winter. This may explain why reasons associated with stalls, e.g., issues with temperature and sunlight, were seldom reported. The reasons that explain fruit and vegetable waste can vary between seasons [12]. We speculate that reasons associated with stalls (i.e., products’ exposure to direct sunlight, excess humidity, poor ventilation, and lack of space for products) and product handling (i.e., products look dehydrated and mechanical damage in products) may occur more frequently in spring or summer. Therefore, we included some recommendations associated with stalls and handling in the tailored informational interventions. During the communication of the intervention to each grocer, special emphasis was placed on those stalls that did not denote problems associated with the physical conditions and handling of products. In these cases, the message was that recommendations associated with stalls and handling apply to the spring and summer months.

### 4.2. Tailored Informational Interventions to Reduce Surplus and Waste

Before the intervention, the measured surplus of fruits and vegetables confirmed the overstock issues reported by the grocers. Almost half of the stock of fruits and vegetables (46.2 and 51.5%, respectively) remained unsold. In contrast, the production of avoidable and unavoidable waste was rather low for fruits (0.1% and 1.8%, respectively) and vegetables (0% and 0%, respectively). Therefore, the seemingly large quantity of fruit and vegetable waste in the stalls represents a small proportion of the initial stock. Moreover, when stored in plastic bags and containers, the avoidable or unavoidable waste reached high volumes but little weight. These data highlight the relevance of expressing avoidable and unavoidable waste as a proportion of the initial stock. Previous data showed that an individual retailer (i.e., distribution level) produces large amounts of waste at the same physical location [54]. Nevertheless, a meta-analysis demonstrated that fruit and vegetable waste varies from 0.4% to 7% depending on the type of product, with waste being higher in small stores [55]. This is an important issue because if the percentage of waste is low, grocers may perceive waste as an intrinsic part of selling, as suggested by previous studies on farmers [23]. Nevertheless, Mattsson et al. [54] suggested that even a minor reduction in the percentage of waste can have an impact on lowering the wasted mass. This is particularly relevant for waste management in the fresh food market. Indeed, the total waste of fruits and vegetables before the intervention reached 18 [15,16,17,18,19,20,21,22,23,24] kg per stall. Considering the 56 stalls within the market, there is ~1000 kg/day of waste that needs to be handled. Moreover, fruit and vegetable waste entails greenhouse gas emissions due to the natural decomposition of food [56]. The amount of greenhouse gas emissions depends on the type of food and its disposal. For fruits or vegetables disposed at a landfill (0% CH_4_ capture), there is an emission of 1535 kg CO_2_ equivalent per ton [57]. With these estimations, one day of operation at the fresh food market will result in the emission of 1535 kg of CO_2_ to the environment in the long term. However, if stalls reduced fruit and vegetable waste, the market would manage less waste, thus diminishing the environmental impact.

Although informative interventions are the most reported, their effectiveness is often questioned [30,31]. This is because informative interventions will only have an impact if translated into behavioral changes. In this pilot study, we observed some behavioral changes in the grocers of the intervention group. Their stalls tended to reduce their stock of fruits, thus decreasing fruit surplus after the intervention. The reduction in the excessive stock of fruits is consistent with the recommendations delivered in the tailored informational interventions. As a group, we did not observe the same response for vegetables. Yet, four out of five stalls reduced their vegetable surplus to a higher extent than the control group. Note that the informational interventions were delivered only once during the study. Perhaps delivering the interventions only once was not enough to promote a behavioral change to reduce the vegetable stock. Repeating the delivery of the intervention or including additional interventions may help achieve behavioral change in all grocers. For example, Roe et al. [58] applied effective tailored interventions at the consumption level where the participants of an intervention group, in a 3-day coaching session, identified which behaviors they needed to change.

Stöckli et al. [30] summarized different interventions to prevent food waste at the consumer level, and found that informational interventions complemented with prompts and commitment are more successful. At the consumer level, Simoes et al. [59] also proposed determining the motivations with the greatest potential to promote behavior change, and involving consumers in the co-design of the interventions. Our results suggest that including informational interventions in larger interventions may help identify and solve major issues that grocers face (e.g., lack of refrigeration infrastructure). The co-design of interventions with the grocers and other stakeholders associated with redistribution might promote behavior changes in the long term.

### 4.3. Economic Cost and Nutritional Content of Avoidable Waste

We found a rather low amount of avoidable waste. Consequently, its financial value for fruits was USD 0.4 [0.0–10.7] per stall before the intervention and USD 0.0 [0.0–5.8] per stall after the intervention; for vegetables, it was USD 27.0 [6.8–40.0] per stall before the intervention and USD 16.8 [1.7–35.0] per stall after the intervention. Previous research showed that the annual fruit and vegetable (fresh and tinned) waste at a supermarket chain is higher when expressed relative to mass (kilograms, 31%) than relative to value (GBP, 16%). This highlights the low financial value per kg of product (or waste) among fruits and vegetables [57]. 

To explore the potential economic benefits to grocers of the intervention, we additionally analyzed the change (Δ) in the financial value of avoidable waste for fruits and vegetables. The Δfinancial value of avoidable waste for fruits and vegetables was calculated as After (USD per stall)–Before (USD per stall). The groups (control vs. intervention) had no difference in the ΔFinancial value of avoidable waste for fruits (USD −0.2 per stall [−13.7–0.0] vs. USD 0.0 per stall [−1.9–1.6], respectively; *p* = 0.556, Mann–Whitney U test) and vegetables (USD −5.1 per stall [−72.4–8.7] vs. USD −11.6 per stall [−14.2–2.9], respectively; *p* = 0.73, Mann–Whitney U test). The economic benefits to grocers may be insufficient to encourage them to avoid food waste. Nevertheless, the financial implications are beyond the price of the fruits and vegetables, as handling a high surplus has potential financial implications for grocers. Grocers would handle less cash because they would not sell a significant part of their products. Moreover, they would need to transport and store the surplus, with uncertainty over whether these perishable products would be finally sold. In addition, food waste at stalls also has environmental and social impacts that grocers should be aware of. Overall, our estimation of the economic and nutritional loss of wasted fruits and vegetables is crucial to evaluate new interventions to reduce fruit and vegetable waste and waste management in fresh food markets [22,60].

Avoidable waste of fruits and vegetables also entails a loss of energy and nutrients. Fruits and vegetables have great nutritional value as they are good sources of dietary fiber, vitamin C, and potassium, which are associated with health benefits [61,62]. It is recommended to consume two servings of fruits and three servings of vegetables per day [63]. The loss of dietary fiber, potassium, and vitamin per 100 g of fruit or vegetables wasted is higher compared to other wasted foods [39]. Therefore, fruit and vegetable waste represents a nutritional issue regardless of the amount wasted.

### 4.4. Limitations

Considering the sample size, the study was only powered to detect large differences between groups, such as those in ΔSurplus of fruits. However, the data will be useful for future sample size calculations. Based our current data and considering a Mann–Whitney U test (α = 5%, β = 20%), 3 stalls per group would be required to detect differences between groups in ΔSurplus of fruits (effect size = 3.30); 10 stalls per group for ΔAvoidable waste of fruits (effect size = 1.39); and 211 stalls per group for ΔUnavoidable waste of fruits (effect size = 0.28). As for vegetables, the values would be 7 stalls per group for ΔSurplus (effect size = 1.82); 28 stalls per group for ΔAvoidable waste (effect size = 0.79); and 287 stalls per group for ΔUnavoidable waste (effect size = 0.24). Most probably, the accuracy from the direct quantification—specifically for either fruits or vegetables—allowed us to better reveal the differences between the groups. Another limitation is that all measurements were conducted in winter during the COVID-19 pandemic. Whether similar findings would be obtained in other contexts is unclear. Yet, our results still suggest that informational interventions may be useful to reduce fruit surplus (and probably vegetable surplus if confirmed by larger studies).

## 5. Conclusions

Through a randomized, controlled, pilot study, we have explored the effectiveness of tailored informational interventions to reduce the surplus and waste of fruits and vegetables. We have observed that, before the intervention, about half of the stock of fruits and vegetables ended up unsold (i.e., became surplus), whereas a low percentage became waste. Planning and storage appeared as the major causes of surplus and waste in the fresh food market. By focusing on these causes, we have shown that tailored informational interventions may improve the management of products and, thus, reduce the surplus of fruits. Future studies should test the effectiveness of combining tailored informational interventions with additional interventions in larger groups of grocers, and during longer periods. Interventions might also consider strategies for the management of surplus which may help grocers to improve their business operations. Interventions focused on reducing the source of surplus can comprise: (a) control of the stock to project a purchase–sale stock according to the season; (b) the control of suppliers that emphasizes the purchase and sale of seasonal fruits and vegetables (in-season products do not require prolonged refrigerated storage); and (c) a price reduction policy agreed upon among grocers (e.g., 25% of the initial price one hour before the closing of the operation day). Further interventions focused on waste can involve: (a) collaboration with non-governmental organizations that redistribute waste for animal feeding; and (b) collaboration with the environmental area of the district to allocate waste unsuitable for animal feeding to a composting center.

## Figures and Tables

**Table 1:** Sociodemographic characteristics of grocers.

	Total	Intervention	Control
n	10	6	4
Sex			
Woman	6	5	1
Man	4	1	3
Age (years)	44 (38–54)	40 (31–52)	48 (44–52)
Education (years)	12 (12–12)	12 (12–12)	13 (12–14)

Data represent the median (25th percentile–75th percentile).

**Table 2:** Fruit and vegetable categories and examples.

Category	Examples
Fruits	
Citrus	Lemons, oranges, mandarines, and grapefruits
Pomace	Apples, pears, and quinces
Banana	Bananas
Vegetables	
Leafy	Lettuce, spinach, chard, cabbage, coriander, parsley, arugula, and chicory
Fruits	Tomatoes, cucumbers, pumpkins, and paprika
Stems	Celery
Inflorescence	Broccoli, cauliflower, and artichoke
Bulbs	Onions and garlic
Roots and tubers	Carrots, radishes, sweet potatoes, and potatoes

**Table 3:** Reasons to explain waste before the intervention.

Reason by Category *	All	Group		
		Control	Intervention	*p*-Value ^$^
Citrus				
Planning, n [%]	9 [100%]	3 [100%]	6 [100%]	-
Storage, n [%]	9 [100%]	3 [100%]	6 [100%]	-
Stall, n [%]	2 [22.2%]	1 [33.3%]	1 [16.7%]	1.00
Handling, n [%]	2 [22.2%]	1 [33.3%]	1 [16.7%]	1.00
Pomace				
Planning, n [%]	4 [100%]	2 [100%]	2 [100%]	-
Storage, n [%]	4 [100%]	2 [100%]	2 [100%]	-
Stall, n [%]	0 [0%]	0 [0%]	0 [0%]	-
Handling, n [%]	1 [25%]	1 [50%]	0 [0%]	1.00
Banana				
Planning, n [%]	6 [100%]	3 [100%]	3 [100%]	-
Storage, n [%]	6 [100%]	3 [100%]	3 [100%]	-
Stall, n [%]	0 [0%]	0 [0%]	0 [0%]	-
Handling, n [%]	1 [16.7%]	1 [33.3%]	0 [0%]	1.00
Leafy				
Planning, n [%]	4 [80%]	1 [100%]	3 [75%]	1.00
Storage, n [%]	5 [100%]	1 [100%]	4 [100%]	-
Stall, n [%]	2 [40%]	1 [100%]	1 [25%]	0.40
Handling, n [%]	4 [80%]	1 [100%]	3 [75%]	1.00
Fruits				
Planning, n [%]	8 [88.9%]	3 [100%]	5 [83.3]	1.00
Storage, n [%]	9 [100%]	3 [100%]	6 [100%]	-
Stall, n [%]	2 [22.2%]	1 [33.3%]	1 [16.7%]	1.00
Handling, n [%]	2 [22.2%]	1 [33.3%]	1 [16.7%]	1.00
Stems				
Planning, n [%]	4 [80%]	1 [100%]	3 [75%]	1.00
Storage, n [%]	5 [100%]	1 [100%]	4 [100%]	-
Stall, n [%]	2 [40%]	1 [100%]	1 [25%]	0.40
Handling, n [%]	5 [100%]	1 [100%]	4 [100%]	-
Inflorescence				
Planning, n [%]	5 [100%]	1 [100%]	4 [100%]	-
Storage, n [%]	5 [100%]	1 [100%]	4 [100%]	-
Stall, n [%]	2 [40%]	1 [100%]	1 [25%]	0.40
Handling, n [%]	0 [0%]	0 [0%]	0 [0%]	-
Bulbs				
Planning, n [%]	4 [80%]	1 [100%]	3 [75%]	1.00
Storage, n [%]	5 [100%]	1 [100%]	4 [100%]	-
Stall, n [%]	2 [40%]	1 [100%]	1 [25%]	0.40
Handling, n [%]	3 [60%]	1 [100%]	2 [50%]	1.00
Root and tubers				
Planning, n [%]	5 [100%]	1 [100%]	4 [100%]	-
Storage, n [%]	5 [100%]	1 [100%]	4 [100%]	-
Stall, n [%]	2 [40%]	1 [100%]	1 [25%]	0.40
Handling, n [%]	2 [40%]	1 [100%]	1 [25%]	0.40

* Percentages calculated based on the number of stalls that sold each category of fruits or vegetables. ^$^ Fischer’s exact test, two-sided.

**Table 4:** Recommendations for each pillar used in tailored informational interventions.

Intervention Pillars			
Planning	Storage	Stall	Handling
- Avoid excessive stock to decrease display times and unnecessary handling [42].	- Maintain the cold chain before and after the day of operation [42].	- Do not expose products to direct sunlight, especially in summer [41].	- Avoid mechanical damage to products to extend their postharvest life through a decrease in ethylene [41].
- Sell the potential surplus of fruits and vegetables cheaper (e.g., 25% less). For this, agree with the other grocers so the offer is launched simultaneously [41].	- Keep separate storage for climacteric fruit and vegetables [41].	- Avoid the direct contact of products with the ground [41].	- Reorder stock using the FEFO method, i.e., First to Expire, First to come Out [42].
- Improve surplus management by adhering to redistribution programs [46].		- Maintain a ventilated and shaded environment, and constantly clean the boxes where the products are stored [41].	- Arrange the products in boxes instead of stacked, to prevent the food from being damaged and crushed [42].
- Use the surplus for animal feeding, composting, and/or bioenergy [42].			- Generate a marketing strategy for a standardized quantity of a product (e.g., four units worth CLP 1000) to sell them prepackaged. This minimizes manipulation by consumers and grocers [42].
			- Raise awareness and guide consumers to take responsible actions when choosing products (e.g., to buy food suitable for consumption despite aesthetic issues) [43].

**Table 5:** Descriptive data of the stalls before and after the intervention.

	All (*n* = 9)		Group			
			Control (*n* = 4)		Intervention (*n* = 5)	
	Before	After	Before	After	Before	After
Fruits						
Stock, kg	560 [122–1078]	215 [133–1029]	540 [44–1126]	532 [48–1126]	560 [267–1068]	215 [185–773]
Surplus, %	46.2 [33.3–51.2]	33.3 [20.9–46.9]	38.8 [25.5–51.5]	41.3 [33.3–54.2]	46.2 [39.8–52.6]	22.2 [18.5–37.0]
Unavoidable waste, %	0.0 [0.0–1.0]	0.0 [0.0–0.5]	0.0 [0.0–1.0]	0.2 [0.0–0.6]	0.0 [0.0–1.1]	0.0 [0.0–0.7]
Avoidable waste						
%	0.1 [0.0–0.8]	0.0 [0.0–0.5]	0.4 [0.0–0.8]	0.0 [0.0–0.0]	0.1 [0.0–0.7]	0.5 [0.0–0.8]
Financial value, USD	0.4 [0.0–10.7]	0.0 [0.0–5.8]	0.2 [0.0–14.6]	0.0 [0.0–0.9]	1.9 [0.0–10.7]	1.9 [0.0–10.3]
Energy, kcal	110 [0–3025]	0 [0–1650]	55 [0–4153]	0 [0–248]	550 [0–3025]	550 [0–2915]
Carbohydrate, g	28.0 [0.0–770.0]	0.0 [0.0–420.0]	14.0 [0.0–1057.0]	0.0 [0.0–63.0]	140.0 [0.0–770.0]	140.0 [0.0–742.0]
Fiber, g	4.0 [0.0–117.4]	0.0 [0.0–64.0]	2.0 [0.0–160.8]	0.0 [0.0–9.8]	21.0 [0.0–117.4]	21.0 [0.0–113.0]
Potassium, mg	349 [0–9594]	0 [0–5233]	174 [0–13,171]	0 [0–785]	1744 [0–9594]	1744 [0–9246]
Vitamin C, mg	67 [0–1834]	0 [0–1000]	34 [0–2518]	0 [0–150]	333 [0–1834]	333 [0–1767]
Vegetables						
Stock, kg	435 [308–935]	398 [284–663]	326 [238–744]	322 [169–831]	437 [397–1102]	578 [357–663]
Surplus, %	51.5 [41.3–55.0]	38.0 [27.1–48.4]	41.3 [37.4–54.8]	41.4 [32.9–51.6]	51.6 [50.8–57.8]	27.7 [25.8–48.3]
Unavoidable waste, %	0.0 [0.0–1.3]	0.0 [0.0–7.8]	0.0 [0.0–2.1]	0.0 [0.0–18.6]	0.4 [0.0–1.3]	5.1 [0.0–7.8]
Avoidable waste						
%	1.8 [0.7–5.3]	2.3 [0.4–3.5]	2.6 [0.5–14.1]	1.6 [0.2–3.6]	1.5 [0.7–5.3]	2.7 [0.7–4.1]
Financial value, USD	27.0 [6.8–40.0]	16.8 [1.7–35.0]	20.3 [3.4–97.5]	15.6 [0.9–35.9]	28.4 [13.0–40.0]	16.8 [6.7–35.0]
Energy, kcal	2964 [751–4399]	3059 [190–4019]	2233 [375–10,716]	1720 [95–3943]	3116 [1425–4399]	3116 [741–4722]
Carbohydrate, g	583.0 [147.5–866.0]	602.0 [37.5–791.0]	439.0 [73.8–2109.3]	338.5 [18.8–776.0]	613.0 [280.5–866.0]	613.0 [146.0–929.5]
Fiber, g	250.0 [63.0–370.5]	258.0 [16.0–338.5]	188.0 [31.5–902.5]	145.0 [8.0–332.3]	262.0 [120.0–370.5]	262.0 [62.5–397.5]
Potassium, mg	39,218 [9930–58,199]	40,475 [2514–53,171]	29,540 [4965–141,790]	22,752 [1257–52,166]	41,230 [18,855–58,199]	41,230 [9805–62,473]
Vitamin C, mg	3680 [932–5460]	3797 [236–4989]	2771 [466–13,303]	2135 [188–4894]	3868 [1769–5460]	3868 [920–5861]

Data are median [25th percentile–75th percentile]. There was no difference between control and intervention groups in any variable before the intervention (*p* ≥ 0.190 in all cases, Mann–Whitney U test).

## Data Availability

Data is contained within the article.
